# Preferential performance of Thal fundoplication for gastroesophageal reflux disease: a single institution experience

**DOI:** 10.1007/s00383-020-04804-y

**Published:** 2021-01-03

**Authors:** Daisuke Ishii, Kazutoshi Miyamoto, Masatoshi Hirasawa, Hisayuki Miyagi

**Affiliations:** Department of Surgery, Asahikawa Medical University, 2-1-1, Midorigaoka-Higashi, Asahikawashi, Hokkaido 078-8510 Japan

**Keywords:** Gastroesophageal reflux disease, GERD, Anti-reflux procedure, Nissen fundoplication, Thal fundoplication

## Abstract

**Purpose:**

Nissen fundoplication (NF) is the most commonly used surgical treatment for persistent gastroesophageal reflux disease (GERD). We introduced to the alternative Thal fundoplication (TF) (partial anterior wrapping) in 1998. The purpose of this paper is to review and report on the effectiveness of TF in our department.

**Methods:**

We retrospectively analyzed cases of 281 patients who underwent TF for GERD at our hospital from 1998 to 2019.

**Results:**

Average age, 16.3 ± 18.1 years; average body weight, 21.0 ± 16.0 kg; average operative time, 89.1 ± 43.0 min; average volume of bleeding, 11.6 ± 29.2 g; enteral feeding commenced after an average of 3.4 ± 1.3 postoperative days (PODs), and average postoperative full enteral feeding was 6.3 ± 1.4 PODs. Five patients (1.8%) had Clavien–Dindo classification III or higher; average hospital stay duration was 10.3 ± 6.0 days, with symptom recurrence affecting 17 patients (6.1%).

**Conclusion:**

TF may be an effective and simple treatment for GERD that has few recurrences and avoids complications common to NF, but further studies to compare it with other techniques are needed.

## Introduction

Gastroesophageal reflux (GER) is also found physiologically in healthy people, especially during infancy, with symptoms often improving with age [1]. However, once GER is associated with any additional symptoms or complications, it is defined as Gastroesophageal reflux disease (GERD). These complications range from esophagitis (resulting from refluxed gastric acid disturbing the esophageal mucosa), aspiration pneumonia (due to the influx of reflux into the trachea), asthma attacks to apnea (via the vagus reflex) amongst others. Such symptoms often promote the occurrence of other conditions [2]. Recently, an association between GERD and apparent life-threatening event has been reported [2]. This association is especially common amongst patients with severe motor and intellectual disabilities [3]. As such, GERD is one of the major contributors to morbidity and mortality in these patients.

In the past, childhood GER was thought to be caused by an immature of lower esophageal sphincter (LES), that the sphincter could not properly respond to surrounding pressure stimuli. However, we now know that it is caused by transient relaxation of the LES lower, or, by a decrease in intra-abdominal pressure [4, 5].

Surgical treatment should be considered for patients with persistent GERD that resists alternative medical treatment, or those who have difficulty withdrawing from medical treatment. The most common fundoplication procedures are the Nissen fundoplication (NF) (full posterior wrapping), the Toupet fundoplication (partial posterior wrapping) and the Thal fundoplication (TF) (partial anterior wrapping). Among these, the NF is the most popular [6, 7]. In the 1970s and 1980s, we performed the NF for GERD. However, since we experienced longer operative times for laparoscopic surgery and had many postoperative complications (such as gas-bloat syndrome), we considered alternative surgical procedures. As a result, we introduced the TF technique to our surgical department in 1998 because we thought that this approach was more physiological, brief and less complicated compared to the NF and the Toupet fundoplication. Therefore, we have reviewed our results obtained with this approach. The purpose of this review was to demonstrate the effectiveness of TF and examine its usefulness over NF.

## Materials and methods

### Subject selection

Subjects included 281 patients who underwent TF for GERD at our hospital from 1998 to 2019. Our department has five indicators for the surgical intervention of GERD, including:

1. Vomiting, pneumonia, apnea and discomfort,
2. Other disorders have been ruled out by upper gastrointestinal imaging that also suggested GER,
3. An esophageal pH-monitored regurgitation rate of 4% or more,
4. A modest attempt at other, less invasive treatments and
5. Granting informed consent to participate in the study.

### Quantified variables

Retrospectively considered variables, including operative method (laparotomy, laparoscopic or laparotomy conversion), simultaneous surgeries at the time of TF, age, body weight, height, underlying disease, operative time (exclusive of any time taken to perform simultaneously performed surgeries, such as a gastrostomy), volume of bleeding, time to commencing postoperative enteral feeding, time to achieving postoperative full enteral feeding, length of hospital stay, complications (Clavien–Dindo classification) [8], and recurrence, were obtained from medical records.

### 2.3 Thal fundoplication procedure

**Fig. 1:**
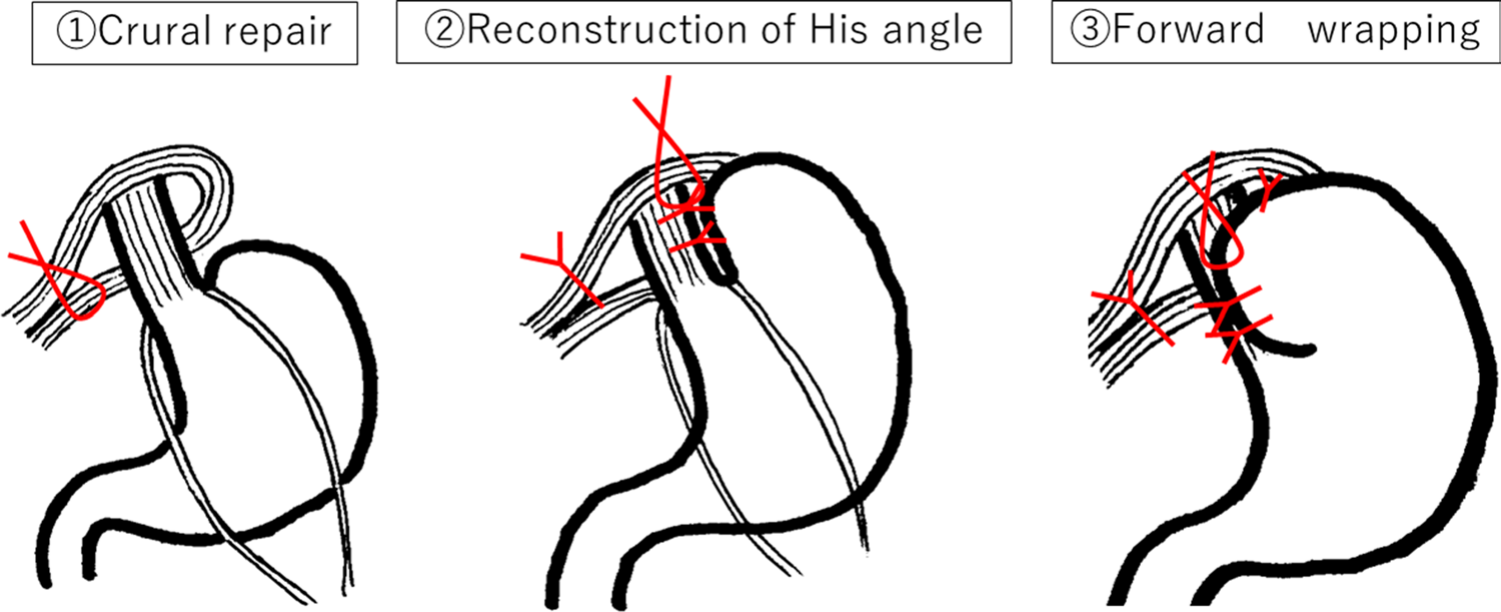
Thal fundoplication ① Crural repair After the abdominal esophagus (at least 3 cm in length) and crura was dissected, the esophageal hiatus was ligated on the dorsal side of the esophagus with non-absorbent thread. This ligation may also be sutured to the posterior esophagus (anchoring suture) to prevent sliding hernia. ② Reconstruction of His angle The left wall of the abdominal esophagus and the fundus of the stomach were sutured to adequately His angulate, and anchoring suture was added to the left crus of the diaphragm. The stomach and the left wall of the esophagus were sutured with two more sutures, and reconstruction of the His angle with a total of three sutures. ③ Anterior Wrapping The greater curvature of the stomach dome was sutured to both the right wall of the abdominal esophagus and the right crus of the diaphragm as an anchoring suture to prevent wrap migration. The stomach and the right wall of the esophagus were sutured with two more sutures, and wrapping it over 180° anterior with a total of three sutures

The procedure of TF was outlined in Fig. 1. The abdominal esophagus (at least 3 cm in length) was taped. After exposing the left and right crura of the diaphragm, the esophageal hiatus was ligated on the dorsal side of the esophagus (Crural repair). Next, the fundus of the stomach was sutured to both the left wall of the abdominal esophagus and left crus of the diaphragm as an anchoring suture to prevent wrap migration. The stomach and left wall of the esophagus were sutured to reconstruct the His angle. Furthermore, the greater curvature of the stomach dome was sutured to both the right wall of the abdominal esophagus and the right crus of the diaphragm as an anchoring suture to prevent wrap migration. The stomach and the right wall of the esophagus were sutured, and wrapping it over 180° anterior [9].

Our inclusion criteria for laparoscopic surgery are listed below:

1. Body weight of 5 kg or more
2. No history of major surgery (minor operations, such as gastrostomy, were not included)
3. No severe cardiorespiratory complications
4. No joint contractures or severe scoliosis

### Statistical analyses

Statistical analyses were performed using EZR (Easy R) (Ver. 1.41) [10]. The statistical software EZR, which is based on R and R commander is freely available on the website (http://www.jichi.ac.jp/saitama-sct/SaitamaHP.files/statmed.html) and runs on Windows (Microsoft Corporation, USA). The results were obtained by employing the one-way analysis of variance (ANOVA) and the Steel–Dwass test. When significant differences were detected, post-hoc analyses were performed using Bonferroni’s corrections. A *p* value of < 0.05 was indicative of statistical significance.

## Results

### Patient demographics and outcomes

Out of all subjects included in this study, there were 263 (93.6%) patients with severe motor and intellectual disabilities. These included 92 patients with cerebral palsy, 21 patients with hypoxic encephalopathy, 18 patients with encephalitis/encephalopathy and 13 patients with other chromosomal abnormalities. Furthermore, 46 subjects reported an esophageal hiatal hernia.

An average follow-up time was 10 years and 3 months. In our sample population, a patient’s average age was 16.3 ± 18.1 years with a mean body weight of 21.0 ± 16.0 kg. Each TF surgery took an average of 89.1 ± 43.0 min, with an average of 11.6 ± 29.2 g of blood loss per patient. Each patient waited an average of 3.4 ± 1.3 postoperative days (PODs) before commencing enteral nutrition, with full nutrition achieved at an average of 6.3 ± 1.4 PODs. Only five (1.8%) patients resulted in a Clavien–Dindo classification of Grade III or higher (i.e., malignant hyperthermia, dysphagia, intestinal obstruction). There were no patients with gas-bloat syndrome among the complications. A patient’s average hospital stay was 10.3 ± 6.0 days, and 17 (6.1%) patients reported symptom recurrence. The outcomes of all patients are summarized in Table 1.

**Table 1:** Results

		All	Open	Lap	Lap → Open
*N*	*n* (%)	281 (100)	69 (24.56)	197 (70.11)	15 (5.34)
Male/Female		173 / 108	43 / 26	123 / 74	7 / 8
Age	years	16.29 ± 18.06	9.99 ± 14.61	18.11 ± 18.30	21.86 ± 23.43
Body weight	kg	20.98 ± 15.98	15.35 ± 12.49	22.98 ± 16.82	21.24 ± 21.20
Height	cm	111.05 ± 34.47	95.73 ± 38.19	116.57 ± 35.36	110.95 ± 118.20
Surgery duration	min	89.11 ± 43.02	59.14 ± 21.87	99.68 ± 44.12	91.67 ± 75.00
Volume of bleeding	g	11.64 ± 29.18	20.33 ± 30.62	7.44 ± 26.35	26.60 ± 6.00
Time to restart feed	PODs	3.41 ± 1.26	3.40 ± 1.64	3.42 ± 1.13	3.27 ± 3.00
Time to full feed	PODs	6.32 ± 1.42	6.37 ± 1.71	6.33 ± 1.34	6.00 ± 6.00
Clavien-Dindo	I	8	3	5	0
	II	19	5	12	2
	III	4	0	3	1
	IV	0	0	0	0
	V	1	1	0	0
Length of hospital stay	days	10.31 ± 6.00	10.28 ± 5.96	10.20 ± 5.54	11.87 ± 8.00
Recurrence	*n* (%)	17 (6.00)	5 (7.24)	12 (6.10)	0 (0.00)

### Technical surgical outcomes

In our study, laparoscopies were performed on the greatest number of subjects (197; 70.1%). Laparotomies were performed on 69 (24.5%) subjects, while laparotomy conversions were only performed on 15 (5.3%) subjects. Laparotomy conversion were due to a poor visual field due to enlargement of the left lobe of the liver and poor pretreatment, giant hiatal hernia, severe adhesions, and bleeding. Average age (years) was laparotomy: laparoscopy: conversion = 9.9 ± 14.6: 18.1 ± 18.3: 21.8 ± 23.43 (laparotomy vs laparoscopy *p* = 0.003), average body weight (kg) was 15.4 ± 12.5: 23.0 ± 16.8: 21.2 ± 21.2 (laparotomy vs laparoscopy *p* = 0.001), average operative time (mins) was 59.1 ± 21.9: 99.7 ± 44.1: 91.7 ± 75.0 (laparotomy vs laparoscopy *p* < 0.001, laparotomy vs conversion *p* = 0.01), average volume of bleeding (g) was 20.3 ± 30.6: 7.4 ± 26.4: 26.6 ± 6.0 (laparotomy vs laparoscope *p* = 0.004, laparoscopic vs conversion *p* = 0.03), average time to start postoperative enteral nutrition (PODs) was 3.4 ± 1.6: 3.4 ± 1.1: 3.3 ± 3.0 (no significant differences), average time to postoperative full enteral nutrition (PODs) was 6.4 ± 1.7: 6.3 ± 1.3: 6.0 ± 6.0 (no significant differences), average hospital stay (days) was 10.3 ± 6.0: 10.2 ± 5.5: 11.9 ± 8.0 (no significant differences) and recurrence rate (%) was 7.2: 5.7: 0.0 (no significant differences).

Some subjects underwent simultaneous surgeries at the time of TF. These included, in order of prevalence, gastrostomy (224 subjects), laryngeal tracheal separation (17 subjects), tracheostomy (14 subjects), diaphragmatic hernia repair (2 subjects), umbilical hernia repair (2 subjects), inguinal hernia repair (2 subjects) and Ladd's operation (2 subjects).

## Discussion

As an intervention for GERD, fundoplication is effectively the surgical reconstruction of the reflux prevention mechanism. It consists of four elements [11]:

1. Securing of the abdominal esophagus,
2. Ligation of the esophageal hiatus,
3. Wrapping of the cardiac part of stomach with the fundus of the stomach and
4. Fixing of the wrap to the diaphragm crura.

Among these, typical methods of wrapping of the cardiac part of stomach include NF (full posterior wrapping), Toupet fundoplication (partial posterior wrapping), and TF (partial anterior wrapping). NF is the most popular [6, 7].

There are few large-scale reports of the TF. Ours is the first report of a single-center study with a large number of cases [12–14]. An anterior partial fundoplication was first described by Thal [15] and later popularized in children by Ashcraft [16].

We report four advantages of preferentially performing TF. First, there is no need to surgically interfere with the short gastric artery and vein. Second, the surgery is brief. Third, herniation of the wrapping valve (wrap migration) does not easily result, and fourth, the prevalence of gas-bloat syndrome is low.

- No need to surgically interfere with the short gastric artery and vein
- Brief surgery and short operative time

In the TF, mobilization of the gastric fundus during fundoplication (partial anterior wrapping) does not require division of short gastric vessels of the spleen and the operation time is short. However, in the NF method, the shoe-shine maneuver or drop test is needed to confirm the free tension of wrap [17]. If the wrap has too much tension, it may be necessary to include a fundus dissection or surgically interfere with the short gastric artery or vein. Under these circumstances, there are risks of splenic bleeds. Three representative papers [18–20] of the Nissen method compared the efficacy and safety of laparoscopies and laparotomies, and we compared our data with respect to TF to this data as well since this report is a single-center, single-operative review (Table 2). On average, our operation time was shorter for both laparoscopic and laparotomy procedures than for the NF. Regarding the length of hospital stay and time to full feed, durations for TF were longer, but we believe that there are due to contributing factors outside the scope of this study as our management method (such as early start of nutrition) that could be improved.

**Table 2:** Comparison with Nissen Fundoplication

	Nissen fundoplication					
References	Knatten et al. [18]		McHoney et al. [19]		Papandria et al. [20]	
Open versus Lap	Open	Lap	Open	Lap	Open	Lap
N	44	44	20	19	21	18
Surgery duration (min)	89 ± 25	150 ± 34	80 ± 5	160 ± 15	89.25 ± 6.25	167 ± 10.5
Length of hospital stay (days)	7.5	7	4.5	5	4.75	4.75
Time to full feed (PODs)	–	–	2	2	–	–
Recurrence (%)	7	37	12	20	4.8	5.6

### Few wrap migration

The NF technique is associated with a wrap disruption/migration rate of 30–40% [21, 22]. In the reconstruction of the angle of His and the anterior wrapping of our TF technique, we always included anchoring sutures with the left and right crura. This is the reason that the wrap migration does not easily result.

### Low prevalence of gas-bloat syndrome

After NF, dysphagia and gas-bloat syndrome due to gastric hyperinflation tended to be more common [11, 12, 23]; however, the partial wrapping of TF resolved these problems. Only two patients needed dilation for postoperative dysphagia. Supporters of a partial wrap believe in its success, claiming that it fixes the problem of GER while leaving the patient with the ability to vomit and burp should the need arise. In addition, NF indicates that the wrap should be short and loose [24, 25]. Loose and short wraps are largely left to the discretion of the surgeon, since there is no clear standard. We believe that partial wraps are more reliable and safer; however, the risks associated with surgical treatment and recurrence of GERD cannot be overlooked. In our data, GERD recurrence rate was 6%. Many reports have shown that there is no significant difference in recurrence rates between the NF, the Toupet fundoplication, and the TF [26–30]. Further, there is no significant difference in recurrence rates between anterior and posterior wrapping or full and partial wrapping. We believe that the recurrence rate was also comparable (Table 2).

In recent years, there have been many reports comparing laparoscopic and laparotomy with the NF [7, 30, 31]. Laparoscopic NF has been shown to be a safe, feasible, and effective surgical procedure alternative to open NF for gastroesophageal reflux in children [7, 30, 31]. We believe that the advantage of laparoscopic TF is similar to the NF. Infants weighing less than 5 kg are excluded from laparoscopic surgery because of the small working space for laparoscopic surgery. Therefore, our results showed that there was a significant difference in age (9.9 ± 14.6: 18.1 ± 18.3 years) and body weight (15.4 ± 12.5: 23.0 ± 16.8) in open surgery compared to laparoscopic surgery for this group. Further, the volume of bleeding during laparoscopic surgery was significantly less than that of open surgery (20.3 ± 30.6: 7.4 ± 26.4 g). Time to start postoperative enteral nutrition (3.4 ± 1.6: 3.4 ± 1.1 PODs), time to postoperative full enteral nutrition (6.4 ± 1.7: 6.3 ± 1.3: PODs) and length of hospital stay (10.3 ± 6.0: 10.2 ± 5.5 days) showed no significant differences between laparoscopic and open surgeries. We were able to complete laparoscopic surgery in 197 (92.9%) of the 212 cases that underwent laparoscopic surgery. Previous studies have reported that, for NF, laparoscopic surgery should be considered an acceptable option for children [30], and we believe the same is true for TF.

## Conclusion

TF did not have a high recurrence rate and was considered effective for treating GERD, but further studies to compare it with other techniques are needed.
